# Echocardiographic assessment of pulmonary hypertension: a hierarchical interpretive framework for clinical decision-making and echo laboratory practice

**DOI:** 10.1186/s44156-026-00138-y

**Published:** 2026-09-28

**Authors:** Aditya Shrivastav, Suhas Tivaskar, Anurag Luharia, Shantanu Gomase, Ranjana Lanjewar

**Affiliations:** 1 School of Allied Health Sciences, Datta Meghe Institute of Higher Education and Research, Sawangi, Wardha, Maharashtra 442001 India; 2 Department of Radiology and Imaging Sciences, School of Allied Health Sciences, Datta Meghe Institute of Higher Education and Research, Sawangi, Wardha, Maharashtra 442001 India; 3 Department of Radiodiagnosis, Datta Meghe Institute of Higher Education and Research, Sawangi, Wardha, Maharashtra 442001 India; 4 Department of Pediatrics, Datta Meghe Institute of Higher Education and Research, Sawangi, Wardha, Maharashtra 442001 India; 5 Department of Cardiovascular & Thoracic Surgery, Datta Meghe Institute of Higher Education and Research, Sawangi, Wardha, Maharashtra 442001 India

**Keywords:** Pulmonary hypertension, Echocardiography, Right ventricular function, Doppler assessment, Risk stratification, Clinical interpretation, Decision-making framework

## Abstract

**Background:**

Echocardiography is central to the evaluation of pulmonary hypertension (PH), yet interpretation of echocardiographic findings remains challenging because non-invasive estimation of pulmonary pressures and right ventricular function is inherently imperfect. Although contemporary guidelines provide structured probability-based assessment, the growing number of available echocardiographic parameters has increased interpretive complexity, particularly in routine echocardiography laboratories where findings must be integrated within diverse clinical contexts.

**Main body:**

This narrative review synthesises contemporary echocardiographic parameters used in PH across key clinical applications, including screening, probability assessment, phenotyping, risk stratification, and longitudinal monitoring. Rather than cataloguing individual measurements, the review examines how pressure estimation, right ventricular structure and function, ventricular interaction, right atrial pressure surrogates, and integrative markers contribute differently according to the clinical question being addressed. Particular emphasis is placed on technical limitations, common sources of measurement uncertainty, and potential discordance with invasive haemodynamic assessment. Building on this synthesis, we propose a hierarchical, decision-oriented interpretive framework designed to support routine echocardiography laboratories by organising existing echocardiographic findings according to clinical purpose and interpretive confidence. The framework emphasises multiparametric integration, internal consistency, contextual interpretation, and transparent communication of uncertainty while complementing established guideline-based probability assessment.

**Conclusion:**

A hierarchical interpretive approach positions echocardiography as a staged component of the broader pulmonary hypertension diagnostic and monitoring pathway rather than as a standalone diagnostic test. By supporting structured reporting, contextual interpretation, and consistent communication of uncertainty, the proposed framework complements existing international guidelines and may facilitate more consistent application of echocardiographic findings within routine clinical practice.

## Background

Pulmonary hypertension (PH) is a progressive haemodynamic disorder associated with substantial morbidity and mortality, in which delayed recognition remains a persistent clinical challenge despite advances in diagnostic strategies [1–3]. Many patients initially present with non-specific symptoms such as unexplained dyspnoea or exercise intolerance, making early identification difficult and often requiring integration of clinical assessment with multimodality investigations [1, 3]. Echocardiography is therefore frequently requested as the first-line imaging investigation when PH is suspected or when unexplained right-heart abnormalities are identified during routine cardiovascular assessment [1, 4, 5].

Although echocardiography is central to the initial evaluation of suspected PH, it cannot establish the diagnosis independently [1, 4]. Current American and European guidelines emphasise that echocardiographic findings must be interpreted alongside clinical history, physical examination, laboratory investigations, cross-sectional imaging where appropriate, and confirmation by right heart catheterisation (RHC) when indicated [1, 4, 6]. Within specialist PH centres, echocardiography therefore represents one component of a broader multidisciplinary diagnostic pathway rather than a standalone diagnostic test [1, 7]. Nevertheless, in routine echocardiography laboratories, echocardiographers frequently encounter technically challenging examinations, discordant imaging findings, or patients with complex comorbidities before comprehensive specialist evaluation has occurred. These circumstances place considerable emphasis on accurate interpretation, appropriate communication of uncertainty, and effective reporting.

The principal challenge in echocardiographic assessment of PH is not the absence of useful parameters but the interpretation of numerous complementary measurements [5, 7, 8], each reflecting different aspects of right ventricular adaptation and pulmonary haemodynamics. Doppler-derived tricuspid regurgitation (TR) velocity [4, 6, 9–11], right ventricular morphology and function, interventricular septal configuration, right atrial pressure (RAP) surrogates, and deformation imaging each provide valuable but incomplete information and are influenced by loading conditions, image quality, and technical acquisition. Consequently, individual measurements should not be interpreted in isolation but integrated within the broader clinical context and the specific diagnostic question being addressed [1, 4, 5].

International guidance from the European Society of Cardiology/European Respiratory Society (ESC/ERS), the British Society of Echocardiography (BSE), the American Society of Echocardiography (ASE), and the European Association of Cardiovascular Imaging (EACVI) provides robust recommendations for probability-based assessment and standardised acquisition of echocardiographic parameters in suspected PH [1, 4, 6, 9]. These recommendations have substantially improved consistency in PH evaluation and remain the foundation of contemporary clinical practice [1, 4]. However, routine echocardiography laboratories continue to face practical challenges when studies are technically limited, multiple parameters appear discordant, or echocardiographic findings must be communicated within diverse clinical pathways. In these situations, interpretation depends not only on individual measurements but also on structured integration of available evidence and transparent recognition of diagnostic uncertainty.

Although RHC remains the reference standard for confirming PH, echocardiography and invasive haemodynamic assessment should be regarded as complementary rather than interchangeable investigations [1, 4]. Differences between echocardiographic estimates and invasive haemodynamics may arise because of technical limitations, physiological variation, loading conditions, or differences in timing between examinations rather than reflecting error alone [5, 10–13]. Understanding these sources of discordance is essential for appropriate interpretation and reinforces the importance of reporting echocardiographic findings within their clinical context.

Numerous reviews have comprehensively described individual echocardiographic parameters used in PH assessment, while international guidelines define structured probability algorithms and recommended measurements [1, 4, 5, 7, 8]. Comparatively less attention has been directed towards organising these parameters according to their practical role within routine echocardiography reporting and their differing relevance across screening, probability assessment, phenotyping, risk assessment, and longitudinal follow-up. As the range of available echocardiographic measurements continues to expand, synthesising existing evidence into an approach that supports consistent interpretation and communication within routine laboratory practice has become increasingly important. Historical studies have long demonstrated that Doppler-derived estimates of pulmonary artery pressure may differ substantially from invasive measurements obtained during RHC, highlighting the need to interpret echocardiographic findings within the broader clinical context rather than as standalone diagnostic evidence [5, 12, 13].

### Aim of this review

The purpose of this narrative review is to synthesise contemporary evidence regarding echocardiographic assessment of PH and to propose a hierarchical, decision-oriented interpretive framework for routine echocardiography laboratories. Rather than introducing new diagnostic criteria or replacing existing guideline-based probability assessment, the proposed framework is intended to complement current recommendations by organising established echocardiographic findings according to their primary clinical purpose, interpretive value, and practical application. By emphasising multiparametric integration, contextual interpretation, and transparent communication of uncertainty, this review aims to support more consistent reporting and facilitate multidisciplinary decision-making across the PH care pathway.

### Methods: narrative synthesis approach

This narrative review was conducted using a structured, non-systematic approach guided by the SANRA principles for narrative synthesis. Literature was identified from major medical databases alongside relevant professional society guidance, with prioritisation of echocardiography-focused studies, consensus documents, and multicentre observational evidence related to PH. The objective was not exhaustive inclusion, but clinically oriented synthesis reflecting echocardiography laboratory practice, interpretive uncertainty, and real-world decision-making across PH care pathways.

## 1. Role of echocardiography in contemporary pulmonary hypertension pathways

Echocardiography occupies a central role throughout the PH care pathway, from the initial evaluation of suspected disease to longitudinal monitoring after diagnosis [1, 4, 5]. Its principal value lies in providing non-invasive structural and functional assessment of the right heart, supporting clinical decision-making before invasive haemodynamic confirmation is considered [5, 7]. Importantly, echocardiography should not be regarded as a standalone diagnostic test but as one component of a comprehensive diagnostic pathway that integrates clinical assessment, laboratory investigations, cross-sectional imaging where appropriate, and RHC when indicated [1, 4].

In patients with suspected PH, echocardiography serves primarily as a screening and probability assessment tool [1, 4]. Current ESC/ERS and BSE recommendations advocate a multiparametric approach that integrates TR velocity with additional echocardiographic markers of right ventricular remodelling, ventricular interaction, and RAP rather than relying on isolated measurements [1, 4]. Because image quality, loading conditions, cardiac rhythm, and technical acquisition influence many echocardiographic parameters, interpretation should always consider the overall pattern of findings rather than individual threshold values [6, 10, 11].

Once PH is suspected or confirmed, echocardiography contributes to disease phenotyping by characterising right ventricular adaptation, ventricular interdependence, atrial remodelling, and coexisting left-sided cardiac disease [7, 8, 14]. These findings assist clinicians in understanding the likely haemodynamic profile and identifying features that may suggest pre-capillary or post-capillary mechanisms while recognising that definitive classification requires integration with the broader clinical assessment and, when appropriate, invasive haemodynamic evaluation [1, 15–17].

Echocardiography also plays an important role in longitudinal follow-up. Serial assessment of right ventricular size, systolic function [18], chamber remodelling, and loading indices enables evaluation of disease progression and response to therapy [18–21]. Although individual echocardiographic measurements remain subject to biological and technical variability, consistent trends observed over time frequently provide more clinically meaningful information than isolated measurements [10, 13, 22]. Standardised acquisition and reporting are therefore essential to maximise the value of serial examinations [4, 6, 9].

Rather than replacing RHC, echocardiography complements invasive haemodynamic assessment by identifying patients who may benefit from further investigation, supporting interpretation of haemodynamic findings, and facilitating ongoing non-invasive surveillance [1, 4]. International guidelines consistently emphasise that clinically meaningful PH assessment depends on multiparametric integration rather than reliance on any single echocardiographic measurement [1, 4, 7].

**Fig. 1:**
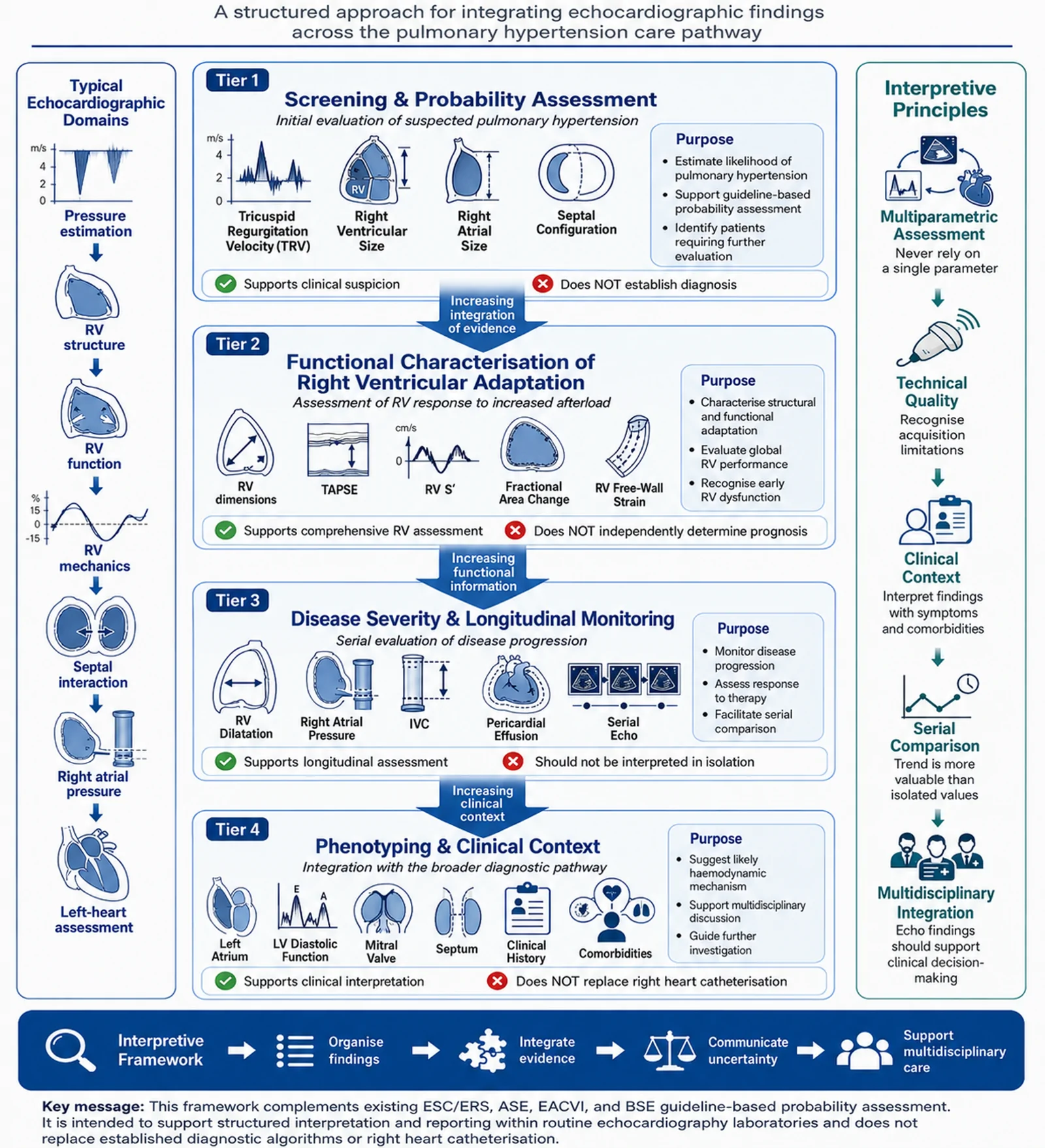
Integration of echocardiography within the contemporary pulmonary hypertension diagnostic and longitudinal care pathway

From the perspective of routine echocardiography laboratories, the clinical purpose of an examination influences how individual parameters should be interpreted. Whether the objective is screening, probability assessment, phenotyping, or longitudinal monitoring, the relative contribution of each echocardiographic marker differs according to the clinical question being addressed. Recognising this distinction provides the rationale for the hierarchical interpretive framework proposed in this review, which is intended to complement existing guideline-based probability assessment by supporting structured interpretation and consistent communication of echocardiographic findings within routine clinical practice (Fig. 1).

### 2. Estimation of pulmonary pressures: strengths and limitations

Doppler-derived tricuspid regurgitation velocity (TRV) remains the cornerstone of non-invasive pulmonary pressure estimation and is the principal echocardiographic parameter used in guideline-based probability assessment of suspected PH [1, 4]. By applying the modified Bernoulli equation, TRV provides an estimate of the right ventricular– RAP gradient from which pulmonary artery systolic pressure may be estimated when RAP is also considered [10, 12]. However, contemporary guidelines emphasise that TRV should be interpreted as part of a multiparametric assessment rather than as an isolated indicator of pulmonary haemodynamics [1, 4].

The strengths of TRV include its wide availability, non-invasive nature, and ability to provide an initial estimate of PH probability in appropriately acquired studies [5, 10]. Nevertheless, accurate measurement depends on the presence of an adequate TR jet, optimal Doppler alignment, and careful signal acquisition [4, 11]. Failure to obtain a measurable TR jet does not exclude PH, particularly in early disease or technically limited examinations, and poor signal quality may substantially reduce measurement reliability [10, 11].

Technical factors remain an important source of variability. Suboptimal acoustic windows, Doppler misalignment, respiratory motion, excessive gain, and imaging artefacts may all result in inaccurate velocity estimation [4, 11]. Even modest deviations from parallel alignment can underestimate peak TR velocity and consequently reduce the estimated pulmonary artery pressure [10]. These limitations should be recognised within echocardiographic reports so that the degree of confidence in pressure estimation is communicated appropriately [4].

Several physiological conditions may also confound interpretation. Elevated TRV may occur in high-output states such as anaemia or fever, or in patients with significant TR, without reflecting primary pulmonary vascular disease [10, 15]. Conversely, severe TR or advanced right ventricular systolic dysfunction may underestimate pulmonary artery pressure because rapid pressure equalisation limits peak jet velocity despite markedly elevated pulmonary vascular resistance [10, 11]. Accordingly, TRV should always be interpreted alongside right ventricular size and function, septal morphology, and other supportive echocardiographic findings rather than in isolation [1, 4].

**Fig. 2:**
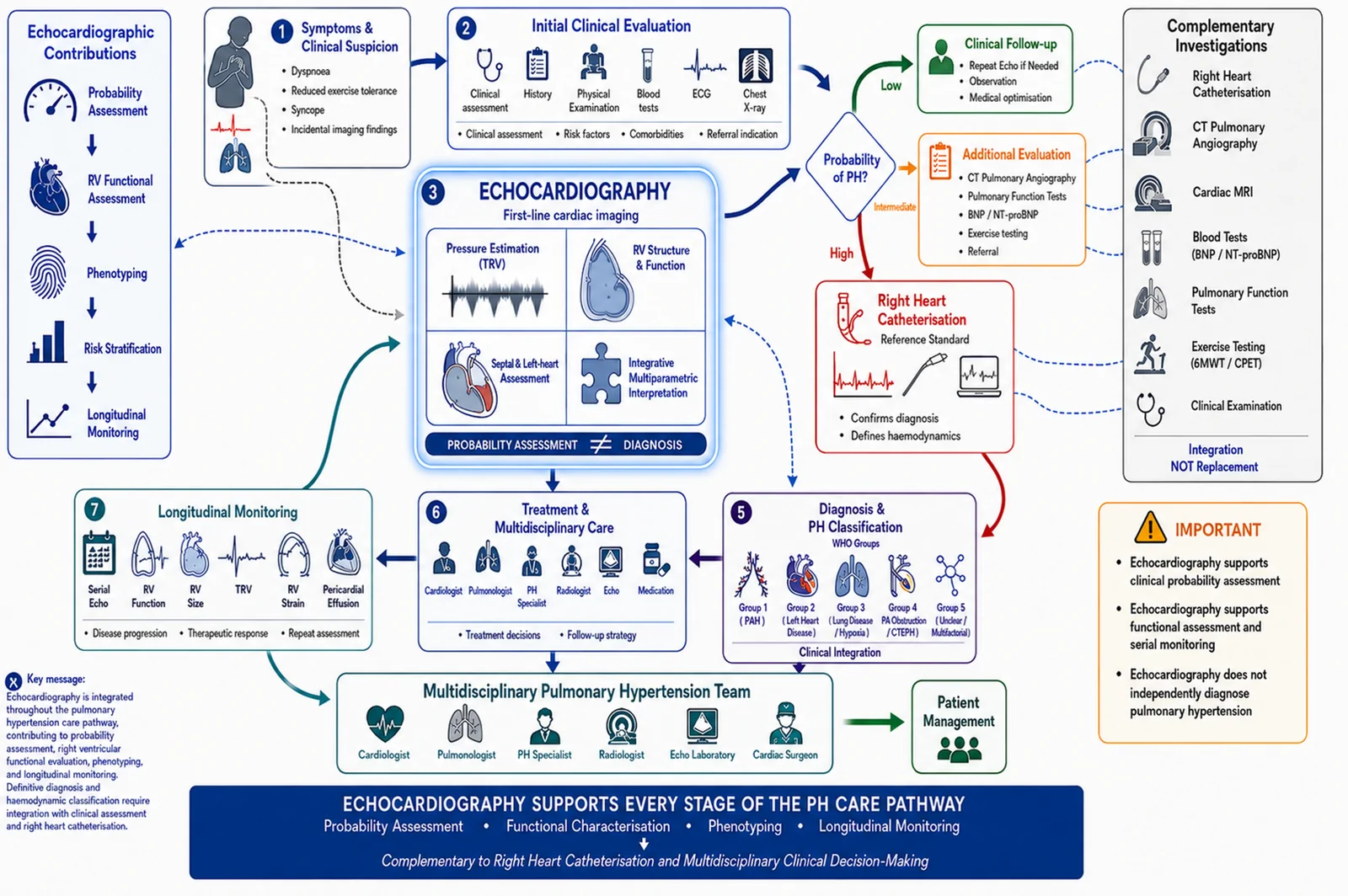
Understanding discordance between echocardiographic and invasive haemodynamic assessment in pulmonary hypertension

An additional consideration is the imperfect agreement between echocardiographic pressure estimates and invasive haemodynamic measurements [12]. Landmark studies, particularly the seminal work of Yock and Popp, first demonstrated that Doppler-derived estimates of right ventricular systolic pressure may differ substantially from simultaneously measured invasive haemodynamic values despite technically satisfactory examinations. Subsequent investigations have confirmed that discrepancies arise from multiple factors, including technical acquisition, estimation of RAP, temporal separation between examinations, changes in loading conditions, and physiological variability rather than systematic failure of echocardiography itself [10, 11, 13, 22]. Recognition of these limitations is essential when interpreting discordant echocardiographic and RHC findings (Fig. 2).

Importantly, echocardiographic estimates of pulmonary artery pressure should not be regarded as direct measurements of pulmonary vascular resistance or mean pulmonary artery pressure [1, 4]. Instead, they represent non-invasive surrogates that contribute to probability assessment and should be interpreted within the broader clinical context [10]. Consistent with current international recommendations, the greatest clinical value of TRV is achieved when integrated with complementary echocardiographic parameters rather than used as an isolated determinant of PH probability [1, 4].

Overall, estimation of pulmonary pressures by echocardiography is best viewed as the entry point to structured multiparametric assessment rather than a substitute for invasive haemodynamic confirmation [1, 4]. Acknowledging the technical and physiological limitations of TRV allows echocardiographers to communicate diagnostic uncertainty more transparently while supporting appropriate clinical decision-making.

### 3. Right ventricular structure and function: beyond single metrics

Assessment of right ventricular (RV) structure and function is fundamental to echocardiographic evaluation of PH, yet remains one of its greatest challenges because of the RV’s complex geometry, marked load dependence, and heterogeneous adaptation to pressure overload [5, 8, 14]. No single echocardiographic parameter adequately characterises RV performance; instead, clinically meaningful assessment requires integration of complementary structural and functional domains that reflect different aspects of RV adaptation [7, 18].

#### RV size and geometry

RV enlargement represents an important marker of chronic pressure overload and adverse remodelling [6, 9]. Measurements of basal and mid-cavity diameter, RV outflow tract dimensions, and qualitative chamber enlargement provide evidence of sustained adaptation to increased afterload rather than acute haemodynamic change [6, 9]. However, RV size remains influenced by imaging plane, foreshortening, and observer variability, while early PH may be present despite normal RV dimensions [4]. Conversely, RV enlargement may also occur in conditions such as congenital heart disease, chronic pulmonary embolism, or isolated volume overload. Consequently, RV size should be interpreted alongside functional indices rather than as an isolated marker of disease severity [23, 24].

#### Longitudinal systolic function (TAPSE and RV S′)

Tricuspid annular plane systolic excursion (TAPSE) and tissue Doppler-derived systolic velocity (RV S′) remain the most widely used measures of RV longitudinal function because they are simple, reproducible, and recommended by contemporary practice guidelines [4, 6]. Nevertheless, both parameters assess motion at a single point of the tricuspid annulus and therefore provide only a partial representation of global RV performance [8, 19]. Their values are influenced by loading conditions, regional dysfunction, pacing, and significant TR, limiting their ability to fully characterise RV contractile function [5, 10]. Accordingly, TAPSE and RV S′ are most informative when interpreted alongside complementary indices or when followed serially over time rather than applied as isolated markers of RV dysfunction [18, 19].

#### Global systolic function (Fractional area change)

RV fractional area change (FAC) provides a broader estimate of systolic performance by incorporating both longitudinal and radial components of RV contraction [4, 6]. Current ASE and BSE recommendations advise that FAC should be measured from the RV-focused apical four-chamber view, rather than the standard apical four-chamber view, because this approach improves visualisation of the RV cavity and enhances measurement reproducibility [4, 6]. Despite offering a more comprehensive assessment than annular motion alone, FAC remains dependent on image quality, accurate endocardial delineation, and consistent acquisition planes [8]. Prominent trabeculations, poor visualisation of the RV apex, or respiratory motion may reduce measurement reliability [6]. Consequently, FAC is best interpreted as a complementary indicator of global RV systolic function rather than a standalone determinant of disease severity.

#### Myocardial deformation imaging (RV strain)

Speckle-tracking echocardiography has expanded the assessment of RV function by quantifying myocardial deformation before conventional indices become abnormal [21, 25]. Current evidence supports RV free-wall longitudinal strain as the preferred deformation parameter because septal strain is substantially influenced by left ventricular mechanics and ventricular interdependence, reducing the specificity of global RV strain for intrinsic RV dysfunction [8, 21]. Compared with conventional longitudinal indices such as TAPSE and RV S′, RV free-wall strain evaluates deformation across the entire free wall and may detect subclinical impairment before overt deterioration in annular motion becomes evident [25].

Despite these advantages, strain imaging remains limited by vendor-specific software, frame-rate dependency, image quality, and variability in endocardial tracking [8]. Furthermore, strain measurements remain load-dependent and should not be interpreted as direct measures of myocardial contractility [14]. Accordingly, RV free-wall strain should be regarded as an adjunctive marker that strengthens multiparametric assessment, particularly when evaluating early RV dysfunction or longitudinal change [18].

#### Integrating RV domains

Each echocardiographic parameter interrogates a distinct component of RV adaptation: chamber size reflects structural remodelling, TAPSE and RV S′ assess longitudinal shortening, FAC estimates global systolic performance, and RV free-wall strain evaluates intrinsic myocardial deformation [7, 18]. Because each parameter has recognised technical and physiological limitations, clinically meaningful interpretation depends on integration rather than prioritisation of individual measurements [1, 4]. Such an approach is consistent with contemporary international recommendations and supports more transparent reporting, improved communication of uncertainty, and more informative multidisciplinary assessment of patients with PH [1] (Table 1).

**Table 1:** Echocardiographic parameters used in pulmonary hypertension: physiological significance, limitations, and interpretive role

Domain	Parameter	Primary physiological information	Major limitations	Interpretive role in routine echocardiography
Pressure estimation	Tricuspid regurgitation velocity (TRV) [1, 11, 12]	Estimates RV–right atrial pressure gradient and contributes to estimation of pulmonary artery systolic pressure	Absent or inadequate TR jet, Doppler angle dependency, severe TR, inaccurate RAP estimation	Primary parameter for guideline-based probability assessment; should always be interpreted with complementary structural and functional findings
RV structure	RV basal diameter and chamber dimensions [6, 9]	Chronic RV pressure overload and remodelling	Two-dimensional geometry, foreshortening, load dependence, limited sensitivity in early disease	Supports assessment of chronic RV adaptation when integrated with functional parameters
RV longitudinal systolic function	TAPSE and RV S′ [6,19]	Longitudinal RV free-wall contraction	Single-point measurement, geometry dependence, load sensitivity, pacing and significant TR may affect values	Useful for serial follow-up and assessment of longitudinal function but should not be interpreted in isolation
RV global systolic function	Fractional Area Change (FAC) [6, 8]	Global RV systolic performance incorporating longitudinal and radial contraction	Requires RV-focused apical four-chamber view, image quality, accurate endocardial delineation, trabeculation	Complements longitudinal indices by providing a broader assessment of RV systolic function
Myocardial deformation	RV free-wall longitudinal strain [21, 25]	Intrinsic RV myocardial deformation and early systolic dysfunction	Vendor variability, image quality, frame-rate dependency, load dependence	Sensitive adjunct for detecting early RV dysfunction and longitudinal change; not recommended as a standalone marker
Ventricular interaction	Interventricular septal flattening or paradoxical septal motion [21, 27]	RV pressure and/or volume overload affecting ventricular interdependence	Influenced by preload, afterload, pericardial constraint and imaging plane	Provides supportive evidence of haemodynamic impact when interpreted alongside other echocardiographic findings
Right atrial pressure	Inferior vena cava (IVC) diameter and respiratory collapsibility [4, 6]	Surrogate estimate of right atrial pressure and systemic venous congestion	Respiratory effort, intrathoracic pressure, patient positioning, technical variability	Supports interpretation of estimated pulmonary pressures and overall right-heart haemodynamics but has limited precision

### 4. Integrative echocardiographic markers and PH phenotyping

Echocardiographic interpretation in PH extends beyond measurement of individual parameters to recognition of patterns that reflect underlying haemodynamic mechanisms and disease phenotype. The objective is not to establish the definitive cause of PH but to integrate structural, functional, and loading-related findings that support clinical hypothesis generation and multidisciplinary decision-making [1, 5, 17]. Such an approach is particularly valuable because many echocardiographic abnormalities are interdependent and acquire greater clinical meaning when interpreted collectively rather than individually [14, 26].

A key objective of phenotyping is distinguishing features suggestive of pre-capillary and post-capillary PH while recognising that overlap frequently exists [1, 15, 24]. Echocardiographic findings favouring pre-capillary PH include disproportionate right ventricular (RV) remodelling, preserved left ventricular systolic function, and the absence of significant left-sided valvular disease [8, 16, 17]. In contrast, post-capillary PH is typically associated with elevated left-sided filling pressures, commonly reflected by left atrial enlargement, left ventricular diastolic dysfunction, or significant mitral valve disease rather than caused by left atrial enlargement itself. These findings provide supportive evidence for the likely haemodynamic mechanism but should not be regarded as definitive without integration with clinical assessment and, where indicated, invasive haemodynamic evaluation [1].

Interpretation may be further complicated by common comorbidities such as systemic hypertension, atrial fibrillation, ageing-related diastolic dysfunction, or mixed cardiac and pulmonary disease [1, 24]. Consequently, transmitral Doppler inflow, tissue Doppler indices, and left atrial size should be interpreted alongside estimated pulmonary pressures to determine whether elevated pulmonary pressures are more consistent with pulmonary vascular disease or secondary to left-heart pathology. Recognition of these confounding factors reduces the risk of inappropriate attribution of elevated pulmonary pressures to pre-capillary PH [1, 9, 17].

Interventricular septal configuration provides an important integrative marker of RV loading. Systolic septal flattening or leftward bowing generally reflects RV pressure overload, whereas persistent diastolic septal flattening suggests a combination of pressure and volume overload [14, 26, 27]. However, septal motion is influenced by preload, afterload, ventricular interaction, and pericardial constraint and should therefore be interpreted in conjunction with other echocardiographic findings rather than in isolation [28].

Assessment of RAP further strengthens integrative interpretation [4, 6]. Inferior vena cava (IVC) size and respiratory collapsibility provide non-invasive surrogate markers of systemic venous congestion and estimated RAP [9]. Nevertheless, these measurements are affected by respiratory effort, intrathoracic pressure, acquisition technique, and patient-specific physiological factors, limiting their precision. Explicit recognition of these limitations within echocardiographic reports improves communication of diagnostic confidence and uncertainty [10, 11].

**Fig. 3:**
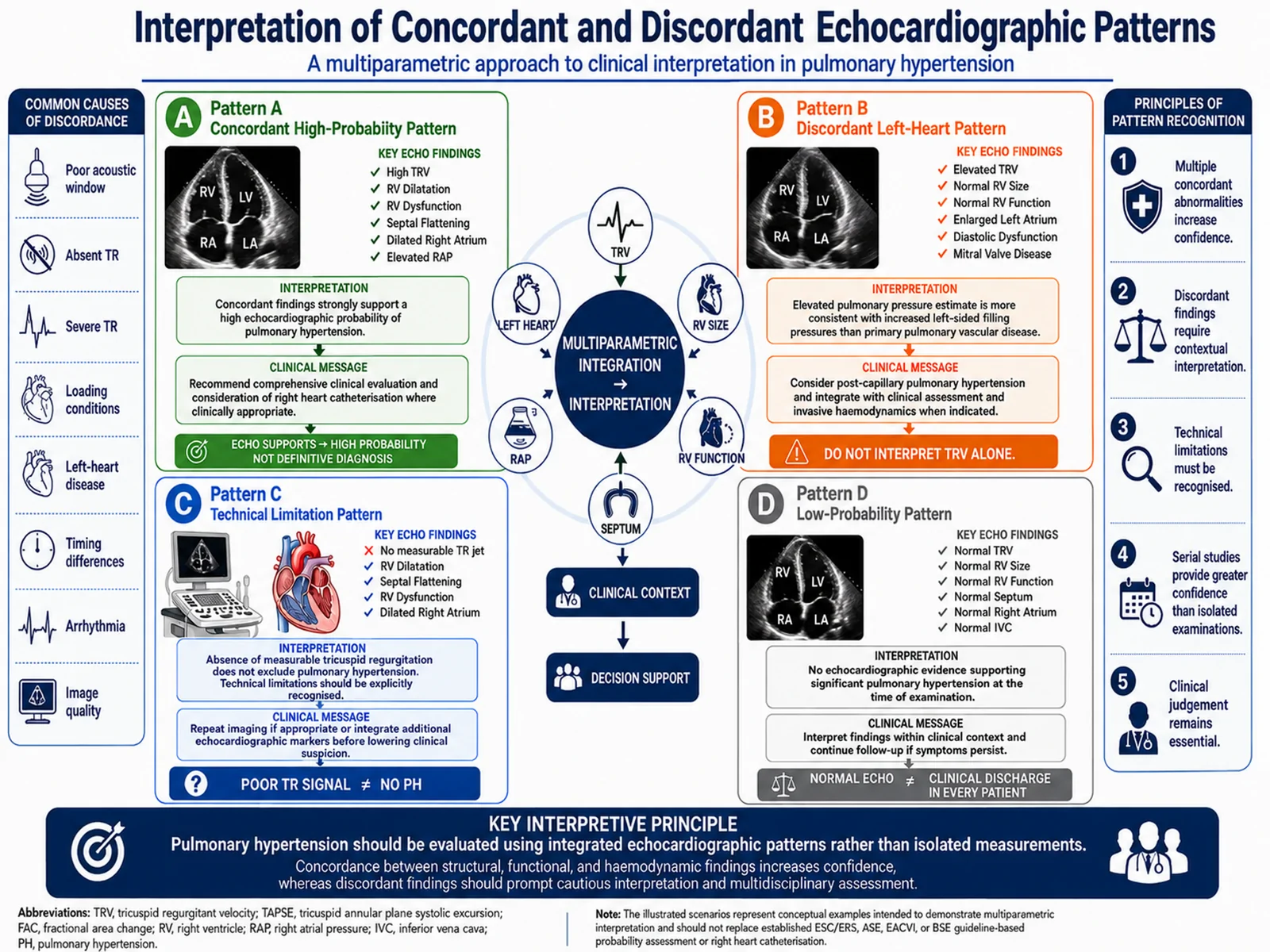
Interpretation of concordant and discordant echocardiographic findings in pulmonary hypertension: a multiparametric pattern-based approach

The greatest value of echocardiographic phenotyping lies in recognising coherent patterns rather than counting abnormal parameters [7, 18, 22] (Fig. 3). For example, elevated TR velocity accompanied by marked RV remodelling, septal flattening, and preserved left-sided structure carries different implications from isolated pressure elevation in the presence of significant left-heart disease [2, 20, 29]. Serial comparison further enhances interpretation by demonstrating evolving patterns of ventricular adaptation over time rather than relying on a single examination [18, 21, 30].

Contemporary ESC/ERS, ASE, EACVI, and BSE guidance all advocate multiparametric integration when assessing suspected PH. Accordingly, integrative echocardiographic assessment should primarily support clinical hypothesis generation, probability assessment, and communication within multidisciplinary teams rather than definitive haemodynamic classification [1, 4, 22]. By combining multiple complementary findings within their appropriate clinical context, echocardiography contributes more effectively to decisions regarding further investigation, specialist referral, longitudinal monitoring, and interpretation alongside RHC while recognising that invasive haemodynamic assessment remains the diagnostic reference standard [1, 7] (Table 2).

**Table 2:** Hierarchical interpretive roles of echocardiography across the pulmonary hypertension care pathway

Clinical context	Primary interpretive role of echocardiography	Principal echocardiographic parameters	Clinical contribution
Suspected pulmonary hypertension	Screening and probability assessment	Tricuspid regurgitation velocity (TRV), RV size, right atrial size, interventricular septal configuration	Supports estimation of PH probability and identifies patients who may require further diagnostic evaluation; not diagnostic in isolation
Intermediate or high probability of PH	Functional characterisation of right ventricular adaptation	TAPSE, RV S′, Fractional Area Change (FAC), RV free-wall strain, RV dimensions	Describes the extent of RV structural and functional adaptation; complements probability assessment rather than confirming PH
Established pulmonary hypertension	Assessment of disease severity and haemodynamic consequences	Right atrial pressure surrogates (IVC), RV dilatation, RV systolic function, pericardial effusion	Supports assessment of disease progression and complements clinical and invasive haemodynamic evaluation
Longitudinal follow-up	Monitoring disease progression and therapeutic response	Serial changes in RV size, RV function, estimated pulmonary pressures, and right atrial pressure surrogates	Emphasises comparison over time, facilitating assessment of treatment response and disease evolution rather than reliance on isolated measurements

Abbreviations: FAC, Fractional Area Change; IVC, Inferior Vena Cava; PH, Pulmonary Hypertension; RV, Right Ventricle; RV S′, Tissue Doppler-derived systolic tricuspid annular velocity; TAPSE, Tricuspid Annular Plane Systolic Excursion; TRV, Tricuspid Regurgitation Velocity

Note: The proposed hierarchy represents the authors'' interpretive framework derived from current ESC/ERS, ASE/EACVI, and BSE recommendations and is intended to complement not replace existing guideline-based probability assessment [1, 4, 6]

### 5. A hierarchical, decision-oriented interpretive framework for echocardiography laboratories

International guidelines from the ESC/ERS, BSE, ASE, and EACVI provide well-established recommendations for the echocardiographic probability assessment of PH and define the parameters that should be incorporated into routine evaluation [1, 4, 6, 9]. Nevertheless, routine echocardiography laboratories frequently encounter technically limited examinations, discordant findings, varying referral indications, and diverse clinical contexts that require interpretation beyond the application of probability algorithms alone [7, 10, 11]. The framework proposed in this review is not intended to replace existing guideline-based probability assessment or introduce new diagnostic criteria. Rather, it is designed as an interpretive framework that organises established echocardiographic findings according to their principal clinical purpose, facilitating structured reporting, communication of diagnostic uncertainty, and integration within multidisciplinary care pathways.

#### Tier 1. Screening and probability assessment

The first tier aligns directly with current international guideline recommendations and focuses on estimating the likelihood of clinically significant PH rather than establishing a definitive diagnosis [1, 4]. Interpretation relies on multiparametric integration of TR velocity (TRV), right ventricular (RV) size, right atrial dimensions, interventricular septal configuration, and other supportive echocardiographic findings [5, 6, 10]. The absence of a measurable TR jet should not exclude PH in technically limited examinations or early disease, while isolated elevation of estimated pulmonary pressure without corroborating structural abnormalities warrants careful review of image quality, loading conditions, and technical acquisition [11, 13, 31].

Within routine echocardiography laboratories, the principal value of this tier is to communicate the overall level of suspicion supported by the examination and identify studies that may require further clinical evaluation rather than to establish a definitive diagnosis.

#### Tier 2. Functional characterisation of right ventricular adaptation

When the probability of PH is considered intermediate or high, the emphasis shifts from probability estimation to assessment of RV adaptation to increased afterload [5, 8]. No individual echocardiographic measurement adequately reflects RV performance; therefore, interpretation integrates RV dimensions, TAPSE, RV S′, FAC, and, where technically feasible, RV free-wall strain [6, 9, 21]. Collectively, these parameters describe the extent of structural remodelling and functional adaptation while recognising the limitations inherent to each measurement [18, 29].

The purpose of this tier is to provide a comprehensive description of RV involvement rather than prognostic classification based on isolated functional indices.

#### Tier 3. Longitudinal assessment and disease severity

Following diagnosis, echocardiography contributes to serial assessment of disease progression and therapeutic response [19, 20, 30]. Progressive RV dilatation, deterioration in systolic performance, increasing RAP surrogates, and development of pericardial effusion reflect evolving haemodynamic burden when interpreted alongside clinical findings and invasive assessment where available [2, 32].

Because echocardiographic measurements demonstrate inherent biological and technical variability, changes observed over serial examinations are frequently more informative than isolated values [10, 13, 22]. Consequently, consistent acquisition methodology and structured reporting are essential for reliable longitudinal comparison [9, 10].

#### Tier 4. Phenotyping and clinical contextualisation

The final tier integrates echocardiographic findings with the patient’s overall clinical context [15, 17, 24]. Differentiation between pre-capillary and post-capillary patterns requires consideration of RV remodelling, ventricular interdependence, left-heart structure and function, atrial remodelling, diastolic indices, and relevant comorbidities [14, 26, 27]. Rather than assigning definitive haemodynamic phenotypes, echocardiography contributes evidence that supports multidisciplinary interpretation and guides consideration of subsequent diagnostic investigations [1].

Importantly, identical echocardiographic findings may have different implications depending on the clinical setting, referral indication, and accompanying structural abnormalities. Contextual interpretation therefore remains fundamental to accurate reporting. The hierarchical organisation of echocardiographic findings according to their principal clinical application is summarised in (Table 3).

**Table 3:** Common pitfalls in echocardiographic assessment of pulmonary hypertension and recommended interpretive approaches

Common pitfall	Why it occurs	Potential clinical consequence	Recommended interpretive approach
Overreliance on tricuspid regurgitation velocity (TRV)	Absent or inadequate TR jet, Doppler misalignment, severe tricuspid regurgitation, inaccurate right atrial pressure estimation	Underestimation or overestimation of PH probability	Always interpret TRV within a multiparametric framework incorporating RV structure, function, and supportive echocardiographic findings
Interpretation based on a single RV functional parameter	RV contraction is complex and no individual parameter completely reflects global RV performance	Misclassification of RV systolic function	Integrate TAPSE, RV S′, FAC, RV free-wall strain (when feasible), and RV dimensions
Failure to recognise left-heart disease	Elevated pulmonary pressures may reflect increased left-sided filling pressures rather than pulmonary vascular disease	Incorrect phenotyping and inappropriate diagnostic pathway	Evaluate left atrial size, diastolic indices, mitral valve disease, and overall left-heart structure together with right-heart findings
Assuming echocardiographic pressure estimates equal invasive haemodynamics	Physiological variability, technical factors, loading conditions, and timing differences between echocardiography and RHC	Apparent disagreement between echocardiography and catheterisation may be misinterpreted as technical error	Interpret echocardiographic findings alongside clinical context and invasive haemodynamic data where available
Interpretation of isolated examinations without longitudinal comparison	Pulmonary hypertension is dynamic and treatment may alter RV adaptation over time	Failure to recognise disease progression or therapeutic response	Prioritise serial comparison using consistent acquisition techniques and standardised reporting
Failure to communicate technical limitations	Image quality, acoustic window, rhythm disturbances, or incomplete datasets may reduce measurement reliability	Overconfidence in echocardiographic interpretation and inappropriate clinical decision-making	Explicitly document technical limitations and communicate the level of interpretive confidence within the echocardiographic report

Sources : Synthesised from Rich et al. Parasuraman et al. Fisher et al., D’Alto et al. Augustine et al. and Amsallem et al. [4, 10, 11, 13, 22, 31]

#### Practical implications for echocardiography laboratories.

The proposed hierarchy is intended primarily as a reporting and interpretive aid for routine echocardiography laboratories. Organising echocardiographic findings according to their principal clinical purpose encourages structured reporting, facilitates communication of diagnostic confidence and uncertainty, and promotes consistent interpretation within multidisciplinary teams. Rather than encouraging exhaustive reporting of individual measurements, the framework emphasises synthesis of complementary findings that directly address the clinical question prompting the examination.

**Fig. 4:**
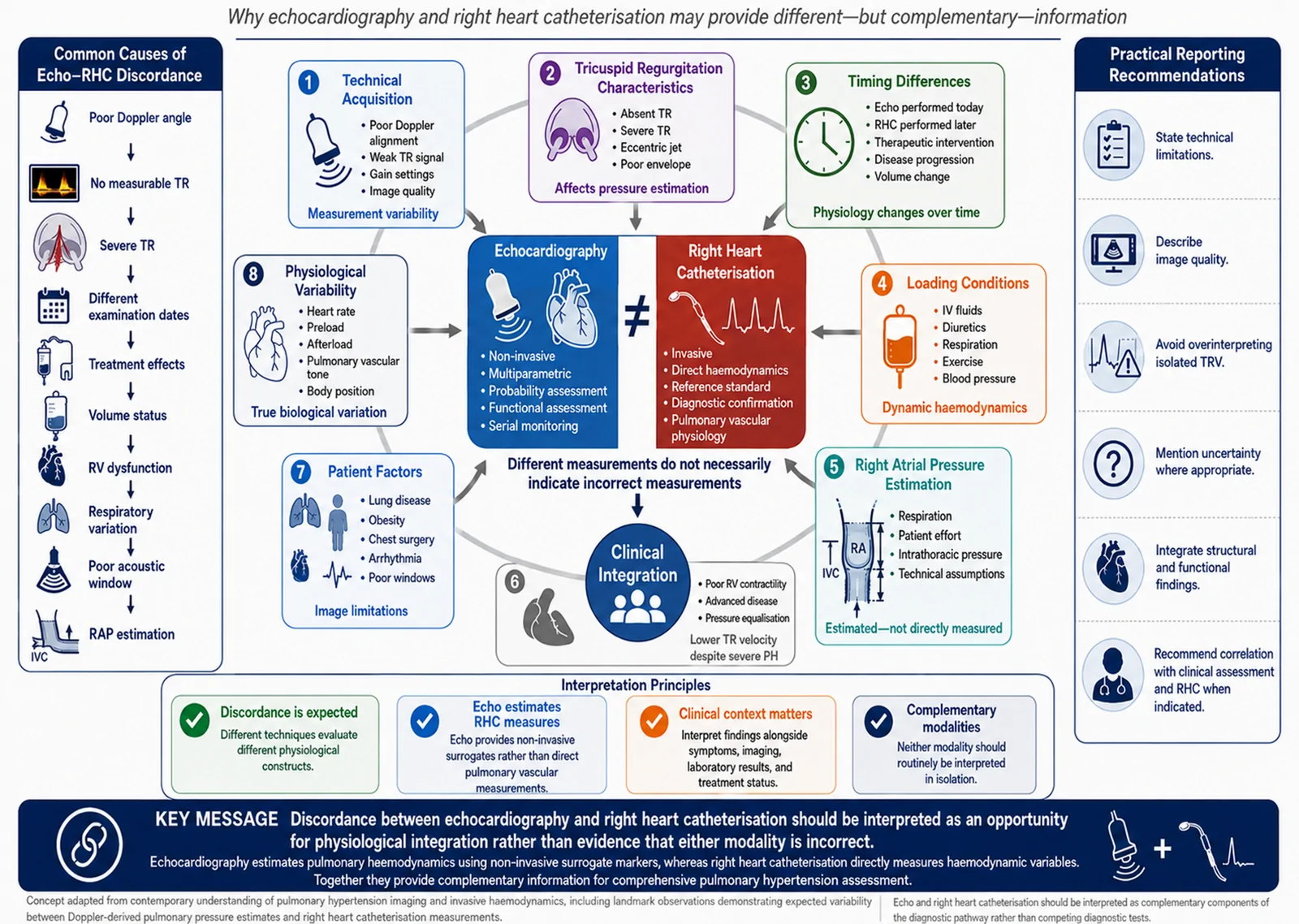
Hierarchical interpretive framework for echocardiographic assessment of pulmonary hypertension across the clinical care pathway

Accordingly, this framework should be viewed as complementary to existing international guideline recommendations rather than as an alternative probability classification system [1, 4]. Its purpose is to support practical implementation of established evidence by providing a structured interpretive approach that reflects the realities of routine echocardiography laboratory practice while recognising both the strengths and limitations of non-invasive cardiac imaging (Fig. 4).

### 6. Current gaps, pitfalls, and future directions

Despite its central role in PH care, echocardiography continues to face important limitations that influence the consistency and clinical interpretation of findings across routine practice. These challenges arise not from a lack of available echocardiographic parameters but from technical variability, operator dependency, and the need to integrate multiple complementary measurements within diverse clinical settings. Recognising these limitations is essential for appropriate interpretation and for identifying realistic opportunities to improve routine echocardiography practice.

Operator dependency remains one of the greatest sources of variability in PH assessment. Measurement of TR velocity, right ventricular (RV) dimensions, FAC, deformation imaging, and inferior vena cava indices all depend on image quality, acquisition technique, and operator experience [31]. Variability in Doppler alignment, endocardial border definition, and image optimisation contributes to inter-observer differences that may influence probability assessment and serial comparison [33]. Standardised acquisition protocols, ongoing quality assurance, and structured training therefore remain fundamental to improving reproducibility across echocardiography laboratories. Increasing use of automated and semi-automated measurement tools may also help reduce inter- and intra-observer variability for selected echocardiographic measurements. However, these tools remain dependent on image quality, appropriate acquisition, and operator oversight and should therefore be considered complementary to, rather than a replacement for, expert echocardiographic interpretation [34].

Although contemporary international guidelines define recommended measurements and probability algorithms, implementation in routine practice remains heterogeneous because laboratories differ in expertise, equipment, patient populations, and referral pathways [35]. Consequently, emphasis may vary between pressure estimation, RV functional assessment, or integrative interpretation, potentially reducing consistency between examinations and across institutions. Structured reporting that explicitly communicates technical limitations, diagnostic confidence, and the clinical question addressed may help improve multidisciplinary communication and longitudinal comparison.

A further challenge is distinguishing measurement from interpretation. Reporting numerous echocardiographic parameters without integrating their collective significance may create an impression of diagnostic certainty while failing to address the underlying clinical question. Conversely, excessive reliance on a single familiar measurement may overlook discordant findings that warrant more cautious interpretation. Additional indices such as the myocardial performance (Tei) index provide complementary information regarding global ventricular function but similarly require interpretation within a broader multiparametric framework rather than as isolated diagnostic markers [36]. Multiparametric synthesis remains the cornerstone of clinically meaningful echocardiographic assessment (Table 4).

**Table 4:** Interpretive echocardiographic domains contributing to multiparametric assessment of pulmonary hypertension

Echocardiographic domain	Primary physiological information	Typical clinical context	Interpretive contribution	Major limitations and caveats
Tricuspid regurgitation velocity (TRV) [1, 11, 12]	Doppler-derived estimate of RV–right atrial pressure gradient	Initial evaluation of suspected PH	Principal parameter for guideline-based probability assessment; interpreted with complementary findings	Dependent on signal quality and Doppler alignment; unreliable in severe tricuspid regurgitation; absent TR does not exclude PH
Right ventricular size and geometry [6, 9]	Structural adaptation to chronic pressure overload	Probability assessment and disease characterisation	Supports recognition of chronic RV remodelling	Two-dimensional geometry, foreshortening, load dependence, limited sensitivity in early disease
TAPSE and RV S′ [6, 19]	Longitudinal RV systolic function	Functional assessment and serial follow-up	Provides simple assessment of longitudinal RV performance	Single-point measurements; influenced by loading conditions, pacing, and regional dysfunction
Fractional Area Change (FAC) [6, 8]	Global RV systolic performance	Functional characterisation	Complements longitudinal indices by incorporating longitudinal and radial contraction	Requires RV-focused apical four-chamber view; dependent on image quality and endocardial delineation
RV free-wall longitudinal strain [21, 25]	Intrinsic RV myocardial deformation	Early RV dysfunction and longitudinal assessment	Sensitive adjunct for identifying subtle RV dysfunction	Vendor variability, image quality, frame-rate dependence, load sensitivity
Right atrial size and pressure surrogates [39, 41]	Chronic right-sided filling pressure and systemic venous congestion	Disease severity assessment	Provides additional haemodynamic context and supports interpretation of RV findings	Influenced by rhythm, respiratory variation, and loading conditions
Interventricular septal configuration [26, 27]	Ventricular interdependence and RV pressure/volume overload	Phenotyping and haemodynamic interpretation	Supports recognition of RV pressure overload and ventricular interaction	Image-plane dependence and preload sensitivity
RV outflow tract flow profile [35]	Pulmonary vascular impedance and RV afterload	Suspected pre-capillary PH	Provides supportive evidence of pulmonary vascular involvement	Heart-rate dependence, technical variability, limited availability
Pericardial effusion [32]	Advanced RV failure and elevated right-sided pressures	Established or advanced PH	Marker of advanced disease severity when interpreted with other findings	Non-specific finding; may reflect alternative pathology
Multiparametric integration [1 , 4, 5]	Combined interpretation of structural, functional, and haemodynamic findings	All stages of PH evaluation	Supports probability assessment, functional interpretation, phenotyping, and longitudinal assessment	Complements but does not replace guideline-based probability assessment or right heart catheterisation

Abbreviations: FAC, Fractional Area Change; PH, Pulmonary Hypertension; RV, Right Ventricle; RVOT, Right Ventricular Outflow Tract; TAPSE, Tricuspid Annular Plane Systolic Excursion; TR, Tricuspid Regurgitation; TRV, Tricuspid Regurgitation Velocity

Sources: Table adapted and synthesised from current ESC/ERS, ASE, EACVI, and BSE recommendations together with representative observational studies cited in the main text

**Fig. 5:**
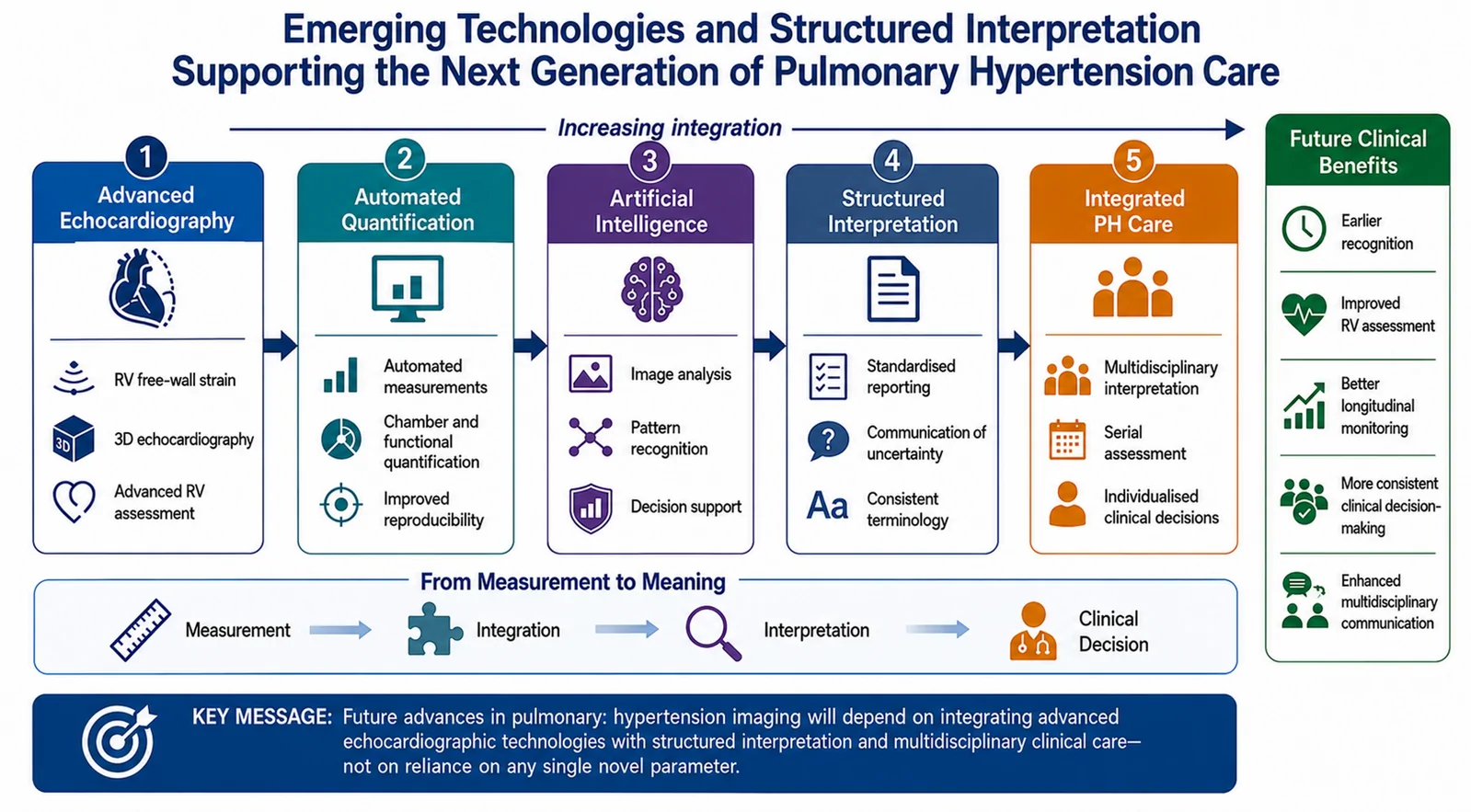
Future directions in echocardiographic assessment of pulmonary hypertension: from conventional imaging to intelligent multiparametric decision support

Future advances are likely to result from a combination of improved imaging techniques and better implementation of existing evidence. Emerging data suggest that RV free-wall longitudinal strain and three-dimensional echocardiography may improve detection of early or subclinical RV dysfunction before conventional indices such as TAPSE or FAC become abnormal [21, 25, 33]. As acquisition techniques, software standardisation, and vendor interoperability continue to improve, these advanced imaging modalities may become increasingly valuable adjuncts to routine PH assessment (Fig. 5), particularly for longitudinal monitoring and early identification of disease progression. Additional echocardiographic markers, including myocardial performance index, right atrial functional assessment, Doppler envelope morphology, and evaluation of RV systolic reserve, have demonstrated promising prognostic value in selected studies but require further standardisation before routine implementation [35–38]. Increasing evidence also supports incorporation of right atrial morphology and function into future risk assessment strategies, while favourable reverse right ventricular remodelling has been shown to accompany successful treatment in selected patient populations [39–41]. Nevertheless, further standardisation and prospective validation are required before widespread implementation.

Equally important are practical improvements within routine echocardiography laboratories. Standardised acquisition protocols, structured reporting templates, explicit documentation of technical limitations, and interpretive frameworks that organise findings according to their clinical purpose may improve consistency without increasing procedural complexity. Such approaches complement existing international guidelines by supporting more transparent communication of uncertainty and facilitating multidisciplinary decision-making.

Ultimately, the principal challenge in echocardiographic assessment of PH is not acquiring additional measurements but interpreting existing information appropriately within its clinical context. Continued advances in imaging technology, combined with improved standardisation, education, and structured interpretation, are likely to enhance the contribution of echocardiography to PH care while preserving its role as a complementary, non-invasive component of the broader diagnostic pathway [18, 21, 37]. This review has limitations. As a narrative review, it does not employ systematic literature search methods or quantitative evidence synthesis. The proposed hierarchical framework has not been prospectively validated and should be regarded as an interpretive model designed to complement existing guideline recommendations. Future multicentre validation studies are required to evaluate its reproducibility, clinical utility, and impact on reporting consistency.

## Conclusions

Echocardiography remains a cornerstone of the evaluation and longitudinal management of PH, providing essential non-invasive information on pulmonary pressure estimation, right ventricular adaptation, haemodynamic consequences, and disease progression. However, its greatest clinical value lies not in any individual measurement but in the structured integration of complementary findings within the appropriate clinical context.

Current international guidelines provide robust recommendations for probability-based assessment and acquisition of echocardiographic parameters. Building upon these recommendations, the hierarchical interpretive framework proposed in this review is intended to support routine echocardiography laboratories by organising established echocardiographic findings according to their primary clinical purpose, including screening, probability assessment, functional characterisation, phenotyping, and longitudinal monitoring. Rather than replacing existing guideline frameworks, this approach aims to facilitate more consistent interpretation, transparent communication of diagnostic uncertainty, and improved multidisciplinary collaboration.

As echocardiographic techniques continue to evolve, future improvements in PH assessment will depend not only on technological advances but also on standardised acquisition, structured reporting, and thoughtful interpretation of multiparametric data. By emphasising context, integration, and reproducibility, this framework seeks to strengthen the practical contribution of echocardiography within contemporary PH care while complementing established evidence-based clinical pathways.

Although developed specifically for PH, the interpretive principles described in this review—including multiparametric integration, structured reporting, and transparent communication of diagnostic uncertainty—may also inform future research into structured interpretive approaches for other echocardiographic applications. Future studies should evaluate whether disease-specific interpretive frameworks can improve reporting consistency, multidisciplinary communication, and clinical decision-making across broader areas of cardiovascular imaging.

## Data Availability

No datasets were generated or analysed during the current study.

## Abbreviations

ASE: American society of echocardiography
AW: Acoustic window
BSE: British society of echocardiography
CMR: Cardiac magnetic resonance
EACVI: European association of cardiovascular imaging
ESC: European society of cardiology
ERS: European respiratory society
FAC: Fractional area change
IVC: Inferior vena cava
LV: Left ventricle / left ventricular
mPAP: Mean pulmonary artery pressure
PA: Pulmonary artery
PAP: Pulmonary artery pressure
PE: Pulmonary embolism
PH: Pulmonary hypertension
PVR: Pulmonary vascular resistance
RA: Right atrium / right atrial
RAP: Right atrial pressure
RHC: Right heart catheterisation
RV: Right ventricle / right ventricular
RVOT: Right ventricular outflow tract
S′: Tissue doppler systolic velocity (Tricuspid annulus)
SANRA: Scale for the assessment of narrative review articles
TAPSE: Tricuspid annular plane systolic excursion
TR: Tricuspid regurgitation
TRV: Tricuspid regurgitation velocity
